# Supplementary material for: Lansoprazole protects hepatic cells against cisplatin-induced oxidative stress through the p38 MAPK/ARE/Nrf2 pathway
Source: PLoS One. 2023 Jun 29;18(6):e0287788. doi: 10.1371/journal.pone.0287788 (PMC10309994; doi:10.1371/journal.pone.0287788)

Raw images

Merge western blot images

Fig. 1A

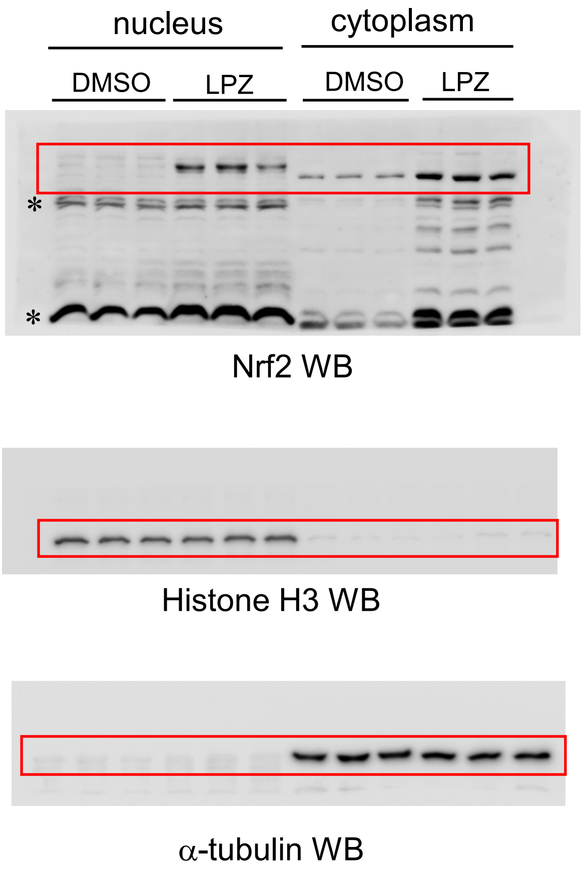

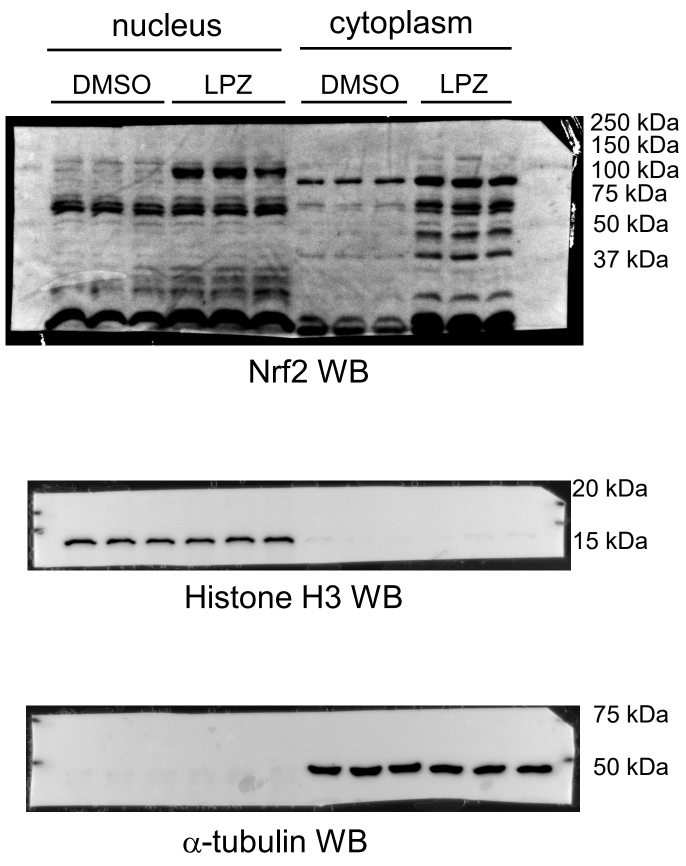

Raw images

Fig. 1E

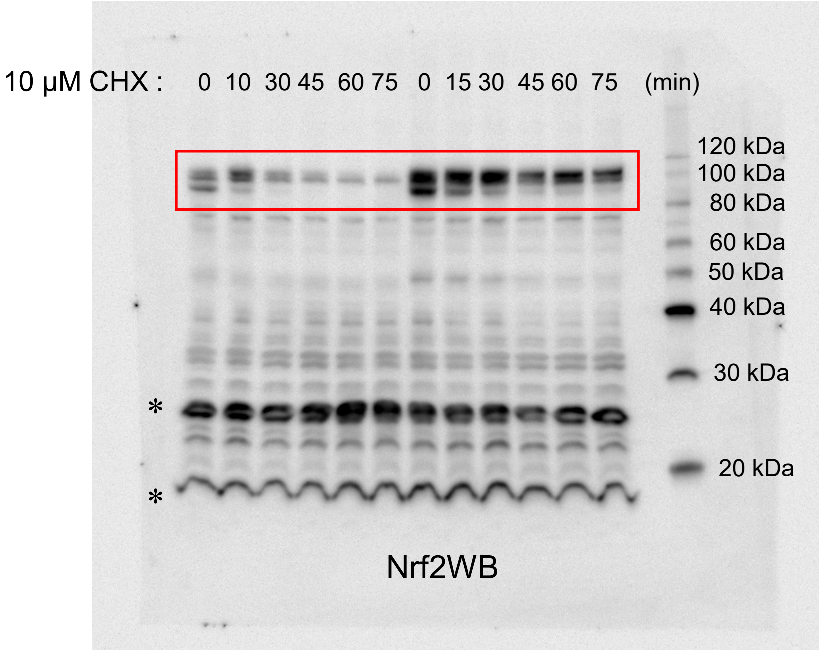

Nrf2WB

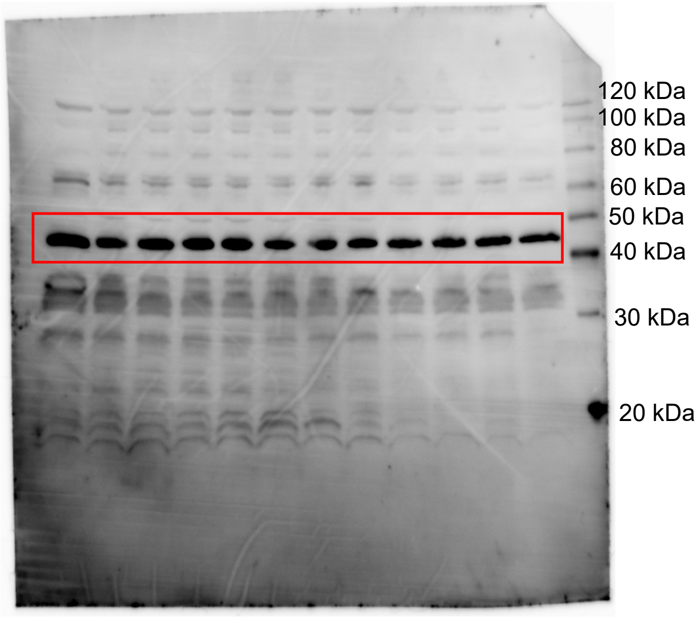

β-actin WB

Fig. 3E

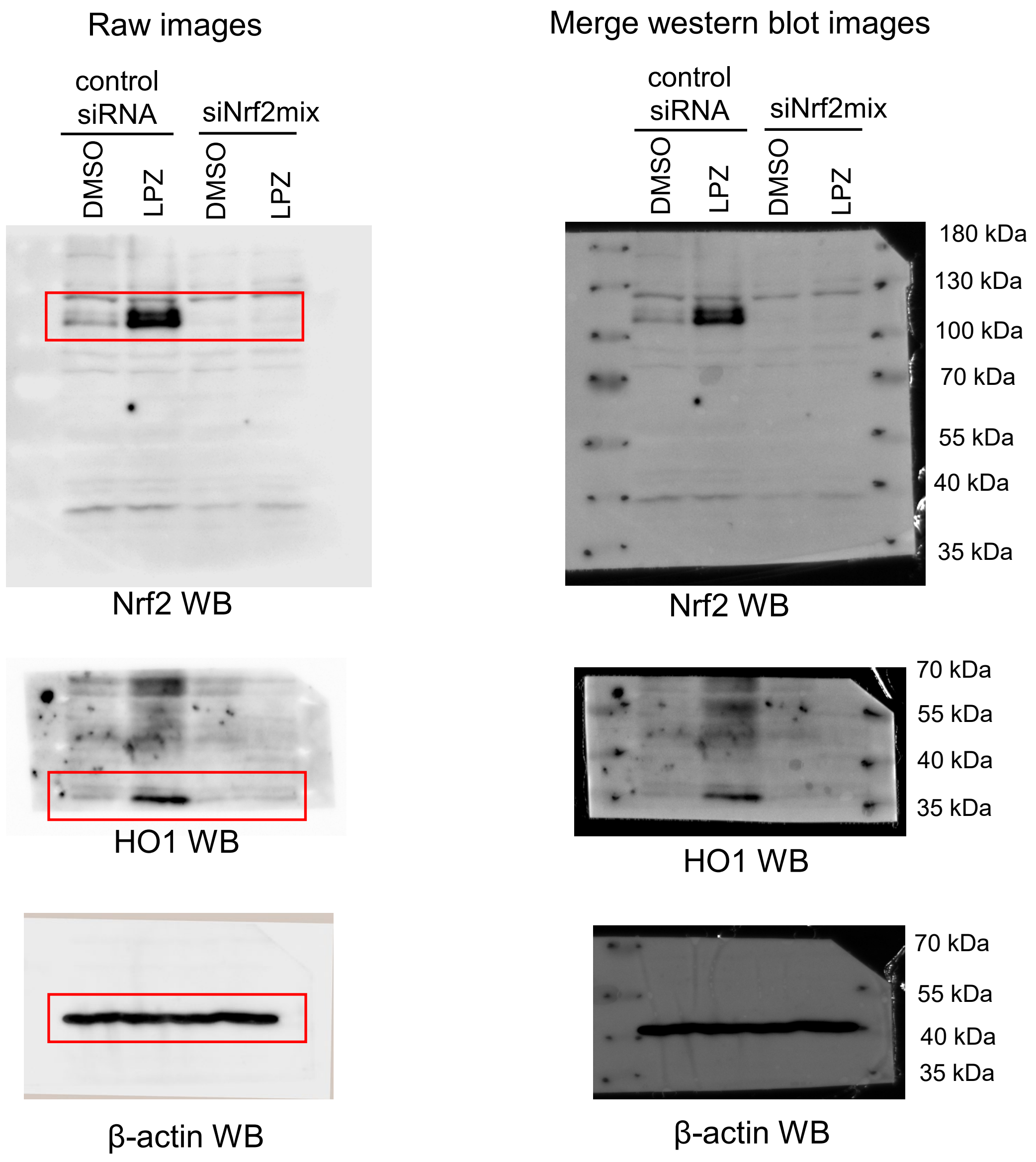

Raw images
control siRNA
siNrf2mix
DMSO
LPZ
DMSO
LPZ
Nrf2 WB
HO1 WB
β-actin WB
Merge western blot images
control siRNA
siNrf2mix
DMSO
LPZ
DMSO
LPZ
180 kDa
130 kDa
100 kDa
70 kDa
55 kDa
40 kDa
35 kDa
Nrf2 WB
70 kDa
55 kDa
40 kDa
35 kDa
HO1 WB
70 kDa
55 kDa
40 kDa
35 kDa
β-actin WB

Raw images

Merge western blot images

Fig. 4A

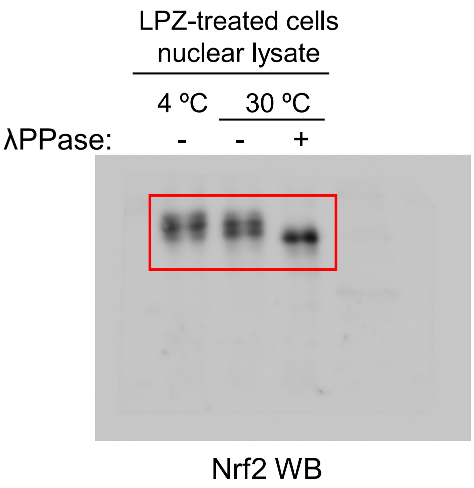

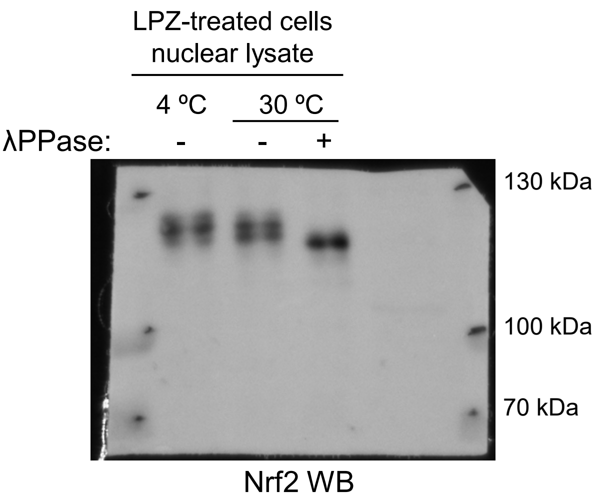

Fig. 4B

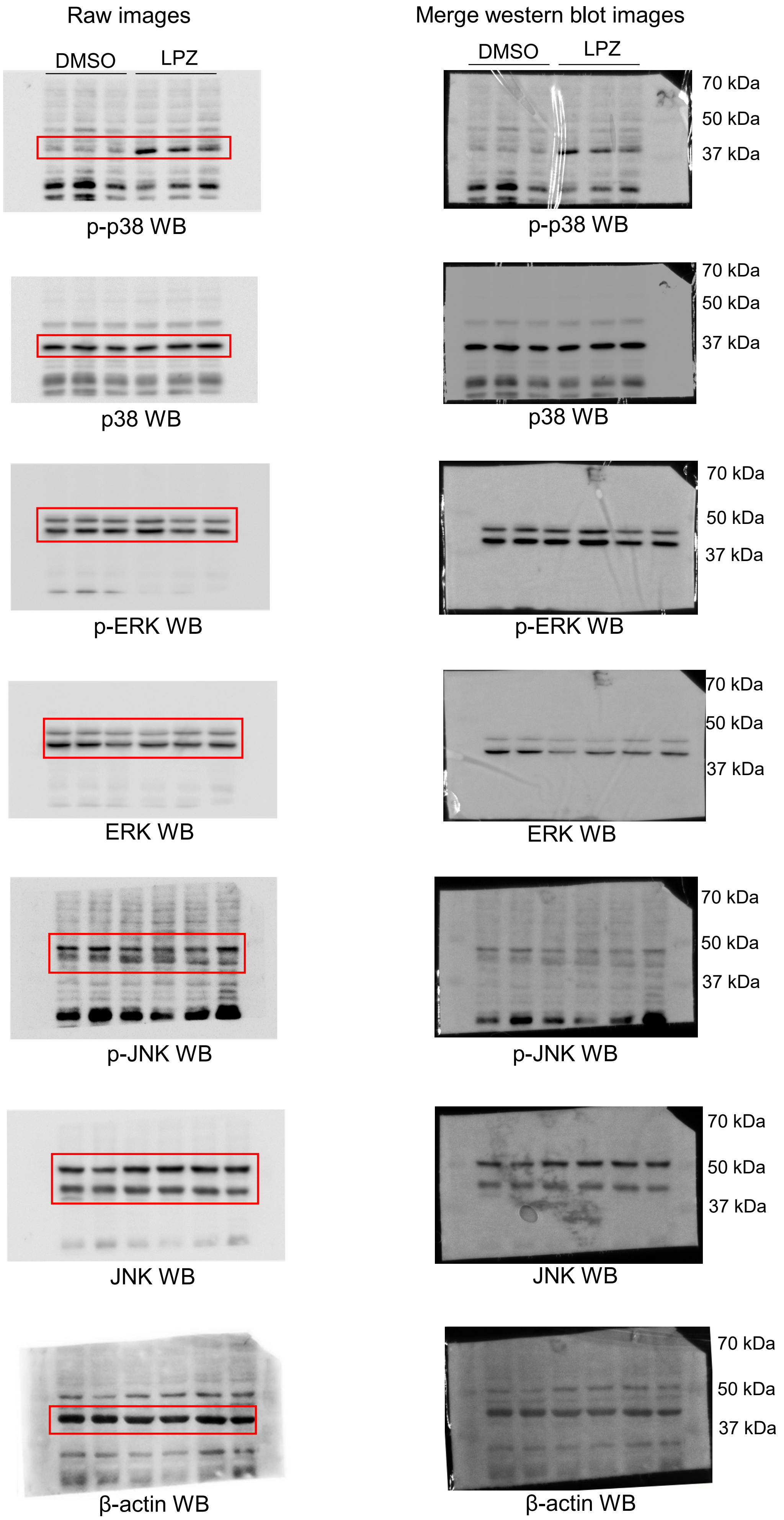

Raw images
DMSO
LPZ
p-p38 WB
p38 WB
p-ERK WB
ERK WB
p-JNK WB
JNK WB
β-actin WB
Merge western blot images
DMSO
LPZ
70 kDa
50 kDa
37 kDa
p-p38 WB
70 kDa
50 kDa
37 kDa
p38 WB
70 kDa
50 kDa
37 kDa
p-ERK WB
70 kDa
50 kDa
37 kDa
ERK WB
70 kDa
50 kDa
37 kDa
p-JNK WB
70 kDa
50 kDa
37 kDa
JNK WB
70 kDa
50 kDa
37 kDa
β-actin WB

Raw images

Merge western blot images

Fig. 5A

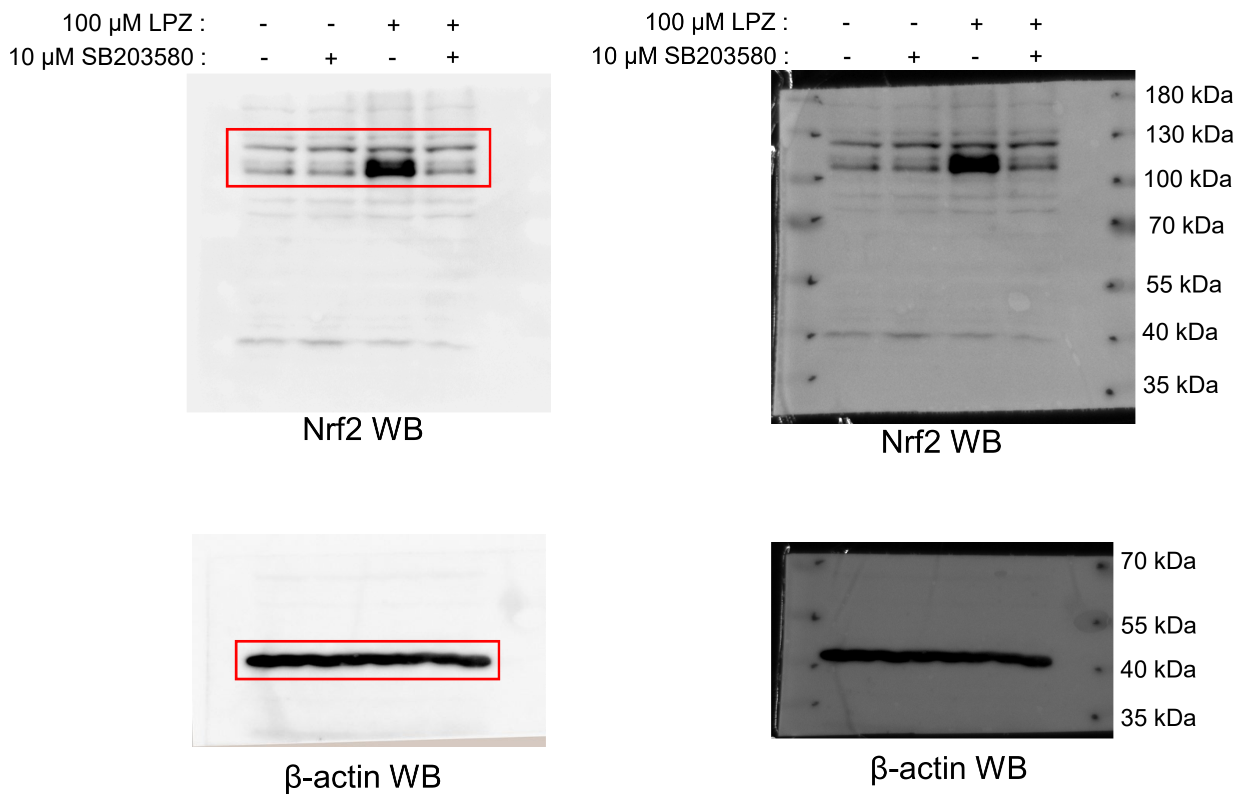

Supplement: S1 Raw images — This file contains all uncropped blot information. The asterisk indicates a nonspecific protein band. (PDF) [file pone.0287788.s001.pdf]
