# Supplementary material for: Lansoprazole protects hepatic cells against cisplatin-induced oxidative stress through the p38 MAPK/ARE/Nrf2 pathway
Source: PLoS One. 2023 Jun 29;18(6):e0287788. doi: 10.1371/journal.pone.0287788 (PMC10309994; doi:10.1371/journal.pone.0287788)
Supplement: Attachment — Submitted filename: PONE-D-22-35612_reviewer report.docx [file pone.0287788.s003.docx]

**Overview:**

Generally, the manuscript presented the novelty of the study in elucidating the potential molecular mechanisms of lansoprazole in cisplatin-induced hepatoxicity in rat hepatic RL34 cells. This manuscript provides an insightful scientific impact on the molecular mechanisms of lansoprazole. The study design and method used were reliable, yet the reporting in methods and results require improvements.

Some recommendations have been suggested as below for revision.

Strengths:

1. This paper presented novel insights on the mechanisms of lansoprazole.
2. The method used was robust and reliable.

Limitations:

1. Poor reporting in methodology and result.
2. Gene expression study with 1 housekeeping gene is less convincing.

Title: Title is too length with too many unnecessary words. Suggest to revise.

**Abstract:**

1. Line 14; 28: Kindly avoid the use of first- and second-person pronouns (i.e. we) in the abstract. Please use active voice and third-person narrative throughout the manuscript.
2. Line 20: Typo for ‘NAD(P)H:1quinone oxidoreductase-1’? It should be written as ‘NAD(P)H quinone oxidoreductase-1’.
3. Method was not mentioned in the abstract.

**Introduction**

1. Line 48: ‘The nuclear Nrf2…’
2. Line 72 - 79: Kindly avoid the use of first- and second-person pronouns (i.e. we) in the abstract. Please use active voice and third-person narrative.
3. Suggest to include the relationship of nrf-2 with p38 and Erk1 in introduction to provide an better overview on the signaling pathway involved and further justified on the choice of antibodies used in Western blotting.

**Methods**

1. Some chemicals or materials only provided with brand name without the city and country of manufacturing, including FBS, DMSO, DMEM, MTS reagent, lansoprazole, RIPA buffer, hygromycin, luciferase assay, anti-rabbit IgG antibody.
2. How many replicates run in cell viability assay (MTS)? How many sets of experiment samples per replicate? Please mention in the method.
3. Line 106: What 96-well plate reader was used? Please specify the brand of equipment along with the city and country.
4. Line 134: CHX abbreviation was used without providing the full term when it was first introduced in the text.
5. Line 140: Version, city and country of ImageJ?
6. Line 151: The amount of total RNA used to reverse-transcribed into cDNA was not mentioned.
7. The experiment setting for gene expression in PCR assay was unclear: volume of reaction mix of PCR, thermal cycling condition, number of replicates, negative control, the validation or optimization of primers, etc.
8. Line 157: Only one housekeeping gene was used (beta actin), which is insufficient and at least two housekeeping genes are require for *in vitro* experiments. Based on MIQE guideline for qPCR, normalization of samples obtained from in vitro experiments can be carried out against a panel (probably two or three) of housekeeping genes whose expression has been shown to be unaffected by experimental conditions. This information must be included in any publication. Refer to MIQE guideline 5^th^ edition published in January 2022. <http://www.econferences.de/miqe-talks/>
9. Line 194: What software was used to run the statistical analyses? Please report the software, version, company, city and country that developed.
10. Number of replicates was unclear in all assays used. Under the figure, it was mentioned three independent experiments were conducted. But the number of replicates in each experiment was unclear.

**Results**

1. Figure 2: What is the indicator for ‘A’? Abbreviations were not provided: SnMP, MTS.
2. Line 252 – 254; line 356 - 359 should be in the discussion rather than results.
3. Figure 3D: The * is missing. What is the indication of the black colour bars vs white and grey bars? From the previous figure (Fig. 2), black bars indicate treatment with LPZ.
4. Line 288: ‘Fig. 4D…’ There is no section D in Figure 4.
5. Figure 4 is not cited in the text.
6. Line 331: ‘Nuclear fractions of the lansoprazole-treated cells were incubated in the presence or absence of λPPase…. (Fig. 5A)’. The figure is incorrectly cited in the text. It should be Fig. 4A. Same for Fig. 5B (line 361), it should be Figure 4B.
7. Legend of Figure 5. The description for (B) and (C) has been wrongly presented. Please revise accordingly.

**Discussion**

1. To include more limitations of the study.
2. To include the future applications or research direction of this study.
